# Cost-effectiveness of adjuvant chemotherapy with uracil–tegafur for curatively resected stage III rectal cancer

**DOI:** 10.1038/sj.bjc.6604666

**Published:** 2008-09-16

**Authors:** A Hisashige, S Yoshida, S Kodaira

**Affiliations:** 1 The Institute of Healthcare Technology Assessment, Tokushima 770-0044, Japan; 2 The Aomori Prefectural Central Hospital, Aomori 030-8553, Japan; 3 Department of Surgery, the Nerima General Hospital, Tokyo 176-8530, Japan

**Keywords:** uracil–tegafur, adjuvant therapy, rectal cancer, economic evaluation

## Abstract

Recently, the National Surgical Adjuvant Study of Colorectal Cancer in Japan, a randomised controlled trial of oral uracil–tegafur (UFT) adjuvant therapy for stage III rectal cancer, showed remarkable survival gains, compared with surgery alone. To evaluate value for money of adjuvant UFT therapy, cost-effective analysis was carried out. Cost-effectiveness analysis of adjuvant UFT therapy was carried out from a payer's perspective, compared with surgery alone. Overall survival and relapse-free survival were estimated by Kaplan–Meier method, up to 5.6 years from randomisation. Costs were estimated from trial data during observation. Quality-adjusted life-years (QALYs) were calculated using utility score from literature. Beyond observation period, they were simulated by the Boag model combined with the competing risk model. For 5.6-year observation, 10-year follow-up and over lifetime, adjuvant UFT therapy gained 0.50, 0.96 and 2.28 QALYs, and reduced costs by $2457, $1771 and $1843 per person compared with surgery alone, respectively (3% discount rate for both effect and costs). Cost-effectiveness acceptability and net monetary benefit analyses showed the robustness of these results. Economic evaluation of adjuvant UFT therapy showed that this therapy is cost saving and can be considered as a cost-effective treatment universally accepted for wide use in Japan.

Colorectal cancer is a major health problem worldwide (Beretta *et al*, 2004; Bernold and Sinicrope, 2006; Monga and O’Connell, 2006). It is the second most frequent cause of cancer death in the United States and most European countries. In Japan, although its number of deaths ranks third, the disease incidence has recently been increasing (Committee of Cancer Statistics, 2005; Ministry of Health, Labor and Welfare, 2006). The prognosis of patients with colorectal cancer has improved steadily over the past several decades, owing to the development of effective therapies (Ragnhammar *et al*, 2001; Beretta *et al*, 2004; Bernold and Sinicrope, 2006; Monga and O’Connell, 2006). In practice, surgery remains the primary treatment modality for localised colorectal cancer. Adjuvant therapy is given in an attempt to eradicate micrometastases and to thereby increase the cure rate after surgical resection.

The natural history of rectal cancer differs from that of colon cancer for epidemiological, anatomical and pathophysiological reasons (Beretta *et al*, 2004; Bernold and Sinicrope, 2006; Monga and O’Connell, 2006). In the adjuvant therapy for stage III rectal cancer, a combined modality therapy consisting of i.v. 5-fluorouracil (5-FU) and radiotherapy is the standard care (Beretta *et al*, 2004; Bernold and Sinicrope, 2006; Monga and O’Connell, 2006). However, the use of chemotherapy has been based on the evidence of postoperative adjuvant chemotherapy in colon cancer, not rectal cancer (Takiuchi, 2006). In rectal cancer, there is only one adjuvant therapy (i.e., the MOF in the NSABP R-01 study), which showed improvement of overall survival (OS) and disease-free survival (DFS), compared with surgery alone as the control (Fisher *et al*, 1988). The optimal timing of adjuvant therapy and the combination of agents continue to evolve. As with in colon cancer (André *et al*, 2004; Twelves *et al*, 2005; Bernold and Sinicrope, 2006; Lembersky *et al*, 2006; Monga and O’Connell, 2006), trials of the combinations of several active drugs, such as oral fluoropyrimidines (i.e., uracil–tegafur (UFT) and capecitabine), oxaliplatin and irinotecan, are under way (Bernold and Sinicrope, 2006; Monga and O’Connell, 2006).

As to radiotherapy, a meta-analysis confirmed that postoperative adjuvant radiotherapy significantly reduced local recurrence, but not overall and cancer-specific mortality (Colorectal Cancer Collaborative Group, 2001). In contrast, meta-analyses have shown that preoperative radiotherapy reduces local recurrence, and overall and/or cancer-specific mortality (Cammà *et al*, 2000). However, randomised controlled trials (RCTs) included in these meta-analyses all started before the broad introduction of total mesorectal excision (TME). The RCT of TME with or without preoperative radiotherapy showed that there was no difference in OS and DFS, except for local recurrence (Kapiteijn *et al*, 2001). Preoperative chemoradiotherapy, as compared with postoperative chemoradiotherapy, decreased local recurrence, but improved neither OS nor DFS (Sauer *et al*, 2004). Moreover, preoperative short-term radiotherapy was suggested to cause delayed adverse reactions (Peeters *et al*, 2005). Therefore, preoperative adjuvant radiotherapy or chemoradiotherapy for rectal cancer should also be carefully evaluated.

In Japan, in contrast to Europe and the United States above, postoperative adjuvant chemotherapy using oral fluoropyrimidines such as UFT, without radiation, has been the primary treatment, mainly because of excellent outcomes of TME with lateral pelvic lymphadenectomy (Takiuchi, 2006). The local recurrence rate in this extended TME was estimated to be lower than simple TME (Akasu and Moriya, 1997; Mori *et al*, 1998). Although individual RCTs comparing oral fluoropyrimidines with surgery alone showed improvement in both DFS and local recurrence in rectal cancer, but not in OS (Kodaira *et al*, 1998; Kato *et al*, 2002), meta-analyses of RCTs related to fluoropyrimidines in the 1980s showed significant benefits in both DFS and OS (Sakamoto *et al*, 1999; Meta-Analysis Group, 2004). In contrast, there has been no evaluation on DFS and OS in other therapies including 5-FU/leucovorin (LV) and radiation, in Japan. On the basis of the evidence, UFT is indicated as the first-line treatment of adjuvant chemotherapy after surgery for stage III rectal cancer in Japan (Japan Society for Cancer of the Colon and Rectum, 2005).

Recently, the National Surgical Adjuvant Study of Colorectal Cancer (NSAS-CC) in Japan (Akasu *et al*, 2006), an RCT of UFT adjuvant therapy for stage III rectal cancer, showed remarkable OS and RFS gains, compared with surgery alone. These results may be attributed to a higher dosage of UFT, rigorous surgical procedures and high quality of the clinical trial. The dosage of UFT per day of 600 mg in this study was greater than the 300–400 mg used in previous UFT studies (Kodaira *et al*, 1998; Sakamoto *et al*, 1999; Kato *et al*, 2002; Meta-Analysis Group, 2004). In this study, TME with lateral pelvic lymphadenectomy was chosen as the control treatment.

Under these circumstances of treatment evolution in rectal cancer, patients and physicians have to consider risks and benefits in choosing the best treatment between the different options now available. On the other hand, health-care payers increasingly require evidence of economics in addition to the clinical value, under the severe pressure to health-care expenditure. However, there is far less economic evaluation specifically of adjuvant therapy for rectal cancer than for colon cancer (van den Hout *et al*, 2002). Only one study, related to cost-effectiveness of adjuvant therapy for rectal cancer, has been reported (Norum *et al*, 1997). Our objective was to confirm the cost-effectiveness of adjuvant UFT therapy in Japan, extrapolating these results to a time horizon sufficiently distant to capture all costs and outcomes of relevance. This study would provide basic information on the cost-effectiveness of adjuvant therapy for rectal cancer not only in Japan, but also in other countries indirectly.

## Materials and methods

### Analytical overview

Economic analysis was conducted retrospectively based on the Japanese NSAS-CC (Akasu *et al*, 2006), a multicentre RCT. Patients with completely resected stage III rectal cancer, who underwent standardised TME with selective lateral pelvic lymphadenectomy, were randomly assigned to either oral UFT (400 mg m^−2^ per day) for 1 year (*n*=139) or surgery alone (*n*=135).

As a type of economic analysis (Drummond *et al*, 2005), a cost-effective analysis was performed. Incremental costs and effectiveness of adjuvant UFT therapy compared with surgery alone were evaluated. According to the effectiveness measure used (i.e., life-years gained and quality-adjusted life-years (QALYs) gained), incremental cost-effectiveness ratios (ICERs) were calculated based on cost per life-year gained and cost per QALY gained, respectively. In a narrow meaning, the former is termed cost-effectiveness analysis, whereas the latter is termed cost–utility analysis.

The payer of National Health Insurance in Japan was adopted as a perspective of economic analysis (Drummond *et al*, 2005). Therefore, for costs, direct medical-care costs (e.g., costs of tests, drugs, health-care personnel and so on) were examined, whereas indirect costs (e.g., time costs or production loss among patients and their families) were not considered. As a time horizon for evaluation, three levels of time periods (i.e., observational period (5.6 years), 10-year follow-up and over lifetime) were considered. As the base case analysis, observational period was used, as this period incorporated few assumptions for the evaluation.

### Effectiveness

The results of the NSAS-CC were used as evidence of effectiveness in the economic analysis. The clinical results have been presented in detail elsewhere (Akasu *et al*, 2006). As is shown in Table 1, between the UFT therapy group and the surgery alone group, no statistical differences were observed in age, sex, tumour location or pathological tumour stage. The incidence of adverse events more than grade 3 in the UFT therapy group was higher than that in the surgery alone group. The OS and RFS rates in the UFT therapy group were higher than those in the surgery alone group (Akasu *et al*, 2006).

**Figure 1:**
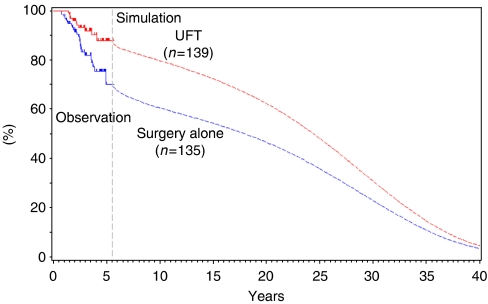
Observed survival curve and extrapolated survival estimate.

Using patients’ data, OS and RFS were estimated by the Kaplan–Meier method, up to 5.6 years from randomisation. Beyond the observation period of 5.6 years, OS was simulated using the Boag model (Boag, 1949) combined with the competing risk model (Gross and Clark, 1975; Maetani *et al*, 2004) (Figure 1). Commonly used methods for extrapolating the survival data beyond the observation include Weibull, Gompertz, exponential, log-normal and generalised gamma distributions (Lee and Go, 1997). However, there is no explicit standard for deciding the optimal method. In this analysis, the Boag model combined with the competing risk model was used. This model consisted of two components: the disease-specific survival curve and the disease-independent survival curve. Although the former was simulated by a log-logistic model, the latter was simulated by the survival curve of the general population matched for age and sex of the subjects. Instead of the log-normal model adopted in the original Boag model, the log-logistic model was selected in this analysis, according to Akaike's Information Criteria (Akaike, 1974).

The mean number of life-years and relapse-free life-years for patients in each group was estimated as the area under RFS and OS curves (Willan and Briggs, 2006). In addition, QALYs were estimated from OS and RFS by weighting each survival by utility value for each possible health state. Utility values for the health states were derived from the published study by Ness *et al* (1999). This study was identified by a systematic literature search using MEDLINE and EMBASE and critically appraised. The median utility (25th, 75th percentile) for stage III after surgery and metastasis was 0.75 (0.55, 0.85) and 0.20 (0.00, 0.40), respectively. These utility values were assigned to the health conditions with and without relapse in this analysis. Although the frequency and grade of adverse events due to chemotherapy was relatively low, utility reduction associated with these events was adjusted by the method adopted by Aballéa *et al* (2007).

### Cost

Costs incurred for resources used during trial and subsequent follow-up were estimated from trial data and their extrapolation. Resource utilisation during trial and follow-up was derived from individual patient history data. As observations on many patients are censored in a clinical trial, subsequent costs are unknown. To correct for censoring, the cost history method proposed by Lin *et al* (Lin *et al*, 1997; Willan and Briggs, 2006) was applied. Costs were estimated from the National Health Insurance perspective using the National Health Insurance reimbursement list and drug price in 2005 (Institute of Social Insurance, 2005; Jiho, 2005). Unit costs for chemotherapy (UFT), consultation and diagnostic tests were low, whereas those of imaging tests for follow-up were relatively high. Admission fees were considerably lower than those in the United States.

Resource utilisation for an adverse event treated on an outpatient basis was estimated for individual patients on standard management. The treatment was relatively simple and at a low cost. The cost of a recurrence was estimated according to the type of recurrence, based on patients’ records during observation. The majority of recurrence was at distant sites, and chemotherapy (e.g., FOLFOX4) is recommended as the first-line therapy in the Japanese guidelines (Japan Society for Cancer of the Colon and Rectum, 2005). The costs associated with end-of-life care for rectal cancer were estimated in the same way as for recurrence. Unrelated health-care costs in the later years of life were not included in this analysis (Drummond *et al*, 2005). All costs were converted from Japanese yen to US dollars based on OECD purchasing power parity in 2005 ($1=¥128) (OECD, 2007).

### Discount

Discounting for the time value of money was applied to both costs and effectiveness. In the base case analysis, both costs and effectiveness accruing beyond 1 year were discounted to present values at a rate of 3%, following the recommendations of the US Panel on Cost-Effectiveness in Health and Medicine (Gold *et al*, 1996). However, currently, much debate still surrounds two major points: the underlying discounting model and the differential discount rate for health and cost (Gold *et al*, 1996; National Institute of Clinical Excellence, 2004; Bos *et al*, 2005; Brouwer *et al*, 2005). Therefore, impact of discounting on the results was examined extensively by sensitivity analysis.

### Sensitivity analysis

The uncertainty of the results was explored by stochastic and qualitative sensitivity analyses of important factors (Glick *et al*, 2001; Briggs, 2004; Drummond *et al*, 2005). The impact of uncertainty on the estimated ICER due to the stochastic nature of sampled data was analysed by applying a non-parametric bootstrap resampling technique (i.e., 5000 times) to both costs and effectiveness. Also, cost-effectiveness acceptability and net monetary benefit (NMB) analyses (Glick *et al*, 2001; Briggs, 2004) were performed. A number of qualitative one-way and two-way sensitivity analyses were conducted to explore the impact of alternative parametric assumptions on the results. These included alternative assumptions concerning time horizon, key cost parameters, recurrence rate, utility value and discount rate.

### Budget impact analysis

To estimate the potential impact of introduction of adjuvant UFT therapy, instead of surgery alone, on the National Health Insurance budget in Japan, a budget impact analysis (Trueman *et al*, 2001) was performed. The total annual cost to the National Health Insurance was estimated using the treatment costs for adjuvant UFT therapy and surgery alone in cost-effective analysis. The time horizon considered in this analysis was 5.6 years for observation period of the NSAS-CC. The costs were discounted at 3% of annual rate. The annual number of the resected stage III rectal cancer in Japan was estimated multiplying the annual incidence rate of rectal cancer (Ministry of Health, Labor and Welfare, 2006) by the proportion of the stage III patients who received initial treatment, at the National Cancer Centre in Japan (Committee of Cancer Statistics, 2005).

## Results

### Effectiveness

The mean LYs and QALYs (3% discount rate) in each group are shown in Table 2A. For 5.6-year observation, 10-year follow-up and over lifetime, the mean QALYs for adjuvant UFT therapy were 3.30, 5.43 and 10.68, respectively. Those for surgery alone were 2.81, 4.47 and 8.38, respectively. Adjuvant therapy gained 0.50, 0.96 and 2.28 QALYs (*P*<0.05). The difference in QALYs was larger than that in LYs for the 5.6-year observation, but this pattern was reversed for 10-year follow-up and over lifetime periods.

### Cost

The mean costs (no discounting) per person in each group for the 5.6-year observation are shown in Table 3. The mean total cost per patient was $10 026 in the UFT therapy group and $12 628 in the surgery alone group. The costs of recurrence and end-of-life were the major components in both groups. Although UFT therapy added over $3000 per patient to the ingredient cost of surgery alone, this was offset by the reduction of costs in recurrence and end-of-life of rectal cancer. As is shown in Table 2A, for 5.6-year observation, 10-year follow-up and over lifetime, adjuvant UFT therapy reduced costs (3% discount rate) per person by $2457, $1771 and $1843 compared with surgery alone, respectively. The difference in the 5.6-year observation was statistically significant (*P*<0.05).

### Incremental cost-effectiveness ratio

Uracil–tegafur therapy showed dominance (less costly and more effective). As is shown in Table 2B, ICER for 5.6-year observation, 10-year follow-up and over lifetime was estimated to be −$4969, −$1815 and −$802 per QALY gained, respectively, using the bootstrap method (3% discount rate for both effect and cost). There is little difference between costs per life-year gained and costs per QALY gained.

### Sensitivity analysis

**Figure 2:**
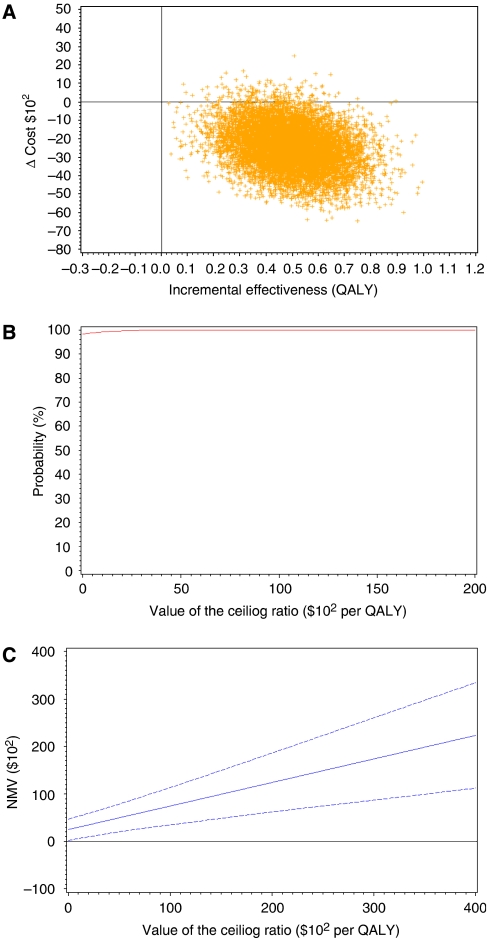
Stochastic sensitivity analyses. (**A**) Incremental cost-effectiveness scatter plot of UFT therapy. (**B**) Cost-effectiveness acceptability curve of adjuvant UFT therapy. (**C**) Net monetary benefit curve of adjuvant UFT therapy with 95% confidence intervals.

The results of a probabilistic sensitivity analysis are shown in Figure 2. Figure 2A shows ICER (cost per QALY gained) scatter plots. More than 95% of the points resided in the southeast quadrant (i.e., more effective and less costly). The cost-effectiveness acceptability cure based on 5000 samples of cost-effectiveness ratio is presented in Figure 2B. Even if additional QALY was valued as 0, the likelihood of UFT therapy being cost-effective was 98%. The NMB curve is shown in Figure 2C. The NMB and its confidence interval (CI) curves did not cross the horizontal axis. This indicates that UFT therapy was beneficial, even if a decision maker was not willing to pay anything for the additional QALY.

A number of qualitative sensitivity analyses are shown in Tables 2B and 4. As to time horizon (Table 2B), from 5.6-year observation to over lifetime, cost-effectiveness ratios were all negative, indicating more benefits and less costs (i.e., dominance).

The two-way sensitivity analysis of discount rate for both costs and effect did not show any change in the dominance of UFT therapy. Incremental cost-effectiveness ratio was lowest (−$5031 per QALY) at a discount rate of 5% for both costs and effectiveness and highest (−$4933 per QALY) without discounting. Incremental cost-effectiveness ratio increased with increase of discount rate of cost, whereas ICER decreased with increase of discount rate of effect.

The results of one-way sensitivity analyses are shown in Table 4. Variations in recurrence rate, utility value, QALYs and the acquisition cost of UFT did not influence the dominance of UFT therapy. On the other hand, with variations of total cost, ICERs varied from −$9695 to $334 per QALY gained. This upper limit of ICER was positive, but at a very low level.

### Budget impact

The annual incidence rate of rectal cancer was 3.23 × 10^4^ and the proportion of the stage III patients was 32.5%. Then, the annual number (95% CI) of the resected stage III rectal cancer was estimated to be 1.05 × 10^4^ (0.91–1.20). The cost reduction per patient was $2603. Therefore, a budget impact converting from surgery alone to adjuvant UFT therapy would reduce current medical expenditure by $27.3 million (95% CI, $23.7 million, $31.2 million) in the first 5.6 years after its adoption.

## Discussion

From the perspective of the National Health Insurance in Japan, this cost-effectiveness analysis of UFT adjuvant therapy for stage III rectal cancer would save health-care costs and improve health outcomes, compared with surgery alone (Table 2). Uracil–tegafur therapy has proved dominant (less costly and more effective). The cost–utility ratio of UFT can be ranked near the top of league table of cost–utility in oncology (Earle *et al*, 2000). The cost savings on the National Health Insurance budget during the observational period would be approximately $27.3 million.

There has been little evidence on economic evaluation of adjuvant therapy for rectal cancer (van den Hout *et al*, 2002). Only one cost-effective analysis of adjuvant therapy for Dukes’ B and C colorectal cancer (Norum *et al*, 1997), of which about two-third consisted of colon cancer, has shown that cost per QALY gained of 5-FU/levamisole was between £4800 and £16,800, compared with surgery alone. This result is highly cost-effective compared with the recent threshold (Jonsson, 2004) for cost-effectiveness. However, cost-effectiveness ratio in our study is much better than that, even though these results are not directly comparable with each other. In the area of rectal cancer, economic evaluations on the standard adjuvant therapy (i.v. 5-FU/radiation) comparing control (i.e., surgery alone), as well as head-to-head evaluation comparing newly emerging with the standard therapy, are urgently needed for health-care decision making. Our study provides basic information in comparing cost-effectiveness of adjuvant therapies for rectal cancer.

To estimate stochastic uncertainty of ICER due to sampling bias in this study, probabilistic sensitivity analyses (Glick *et al*, 2001; Briggs, 2004; Drummond *et al*, 2005) were performed (Table 2, Figure 2). Cost-effectiveness scatter plots showed that CIs of ICER located in the southeast quadrant (more effective and less costly) on the cost-effectiveness plane. Cost-effectiveness acceptability and NMB curves give more information than simple intervals or plots of ICERs mentioned above. Even if a decision maker was unwilling to invest anything at the maximum to achieve additional QALY, the likelihood of UFT therapy being acceptable as cost-effective was 98% (Figure 2B). At the same condition, the NMB curve showed that UFT therapy was beneficial ($2450 per person) (Figure 2C). These results show that the dominant cost-effectiveness of UFT adjuvant therapy is robust. This dominance was shown more sensitive to costs than effectiveness by the scatter plot of ICERs (Figure 2A) and broad one-way sensitivity analysis on both QALY gained and total costs (Table 4).

The time horizon is an important issue to sufficiently capture relevant costs and health outcomes of UFT adjuvant therapy. The observation period of the NSAS-CC, 5.6 years, was limited. Although most costs were incurred mainly in the observational period, life-years gained would continue after it. In this study, a simulation model was used to extrapolate its results. There is a variety of ways for simulation (Lee and Go, 1997), but no uniform methodology is available. In the analysis, we used the Boag model combined with the competing risk model (Maetani *et al*, 2004). In a sensitivity analysis, ICER of the observational period (the base case analysis) was compared with that in the extrapolated periods (i.e., 10-year follow-up and over lifetime). These ICERs were dominant (more effective and less costly) in spite of relative differences in their values.

The key drivers of the dominant cost-effectiveness results of UFT are mainly the savings achieved by reduction of costs related to recurrence and death, which offset the acquisition cost of UFT. After a 4-year follow-up, any recurrence has not been observed in this study. In one-way sensitivity analysis (Table 4), varying recurrence rates between 95% CIs did not have any substantial impact on dominant cost-effectiveness. The other cost driver was the acquisition costs of UFT. Variation in the price of UFT and its standard regimen would be unlikely. Varying acquisition costs of UFT did not have any impact of dominance (Table 4).

Cost-effectiveness analysis using QALYs offers the opportunity to consider both quantity and quality of survival. However, no substantial difference in ICERs was observed between cost per LY gained and QALY gained (Table 2). In addition, the sensitivity analysis on range of utility values for recurrence and non-recurrence revealed no major change in dominant cost-effectiveness (Table 4). In a sizable fraction of cost-effectiveness analysis, utility weighting was indicated not to substantially alter the estimated cost-effectiveness of an intervention (Chapman *et al*, 2004). It is thus suggested that sensitivity analyses using *ad hoc* adjustment or weight from the literature may be sufficient. Our results support this conclusion.

The impact of discounting for the time value of money on the results was examined extensively by two-way sensitivity analyses. Although ICERs were more sensitive to cost discounting than effectiveness discounting, there was no substantial change in dominant cost-effectiveness. The main reason is likely to be that major costs were incurred during the early phase of follow-up and improved survival was realised simultaneously.

There are several limitations in the analysis that should be commented on, and the results should be treated with caution. First, the analysis was based on a small RCT with relatively short-term follow-up (Akasu *et al*, 2006). Therefore, extensive sensitivity analyses were performed to examine this uncertainty. However, large-scale RCTs are crucial to resolving this issue. Second, the UFT adjuvant therapy was the only chemotherapy that showed improvement in OS and DFS compared with surgery alone in Japan. As there is no RCT comparing the standard adjuvant therapy (e.g., 5-FU/radiation) with surgery alone or UFT, it is impossible to directly or indirectly compare these therapies. In future, head-to-head evaluations comparing new emerging therapies (e.g., capecitabine and oxaliplatin) will need to be carried out. Third, the perspective of this analysis is that of a payer for health care, rather than a society. From a societal perspective, the range of costs is broader and includes time costs and travel costs associated with treatment and loss of production due to earlier death. As UFT adjuvant therapy increased OS and decreased recurrence, their cost reduction in both indirect costs and direct costs will offset the costs corresponding to a treatment period.

The issue of generalisability of this study to other countries should be carefully examined, as the UFT adjuvant therapy is the standard in Japan. However, as mentioned in the introduction, in contrast to colon cancer, there is no firm evidence for effectiveness of the western standard therapy (e.g., FU/LV) in rectal cancer. Moreover, given the high treatment costs, substantial toxicity and relatively limited efficacy of the fast-changing chemo- and immunotherapeutic combinations for colorectal cancer, examination of cost-effectiveness studies should be conducted on a routine basis along with determination of clinical benefits (Jansman *et al*, 2007). Uracil–tegafur has been approved and utilised in 31 countries from Europe and Canada to Asia, excluding the United States. Recently, several studies of combination therapies of UFT/LV in metastatic colorectal cancer have shown efficacy (Bennouna *et al*, 2006; Bajetta *et al*, 2007). Therefore, the results of this study indicating effectiveness and cost-effectiveness of UFT adjuvant therapy in rectal cancer may be useful as a reference case for the direct or indirect future examination internationally.

## Figures and Tables

**Table 1:** Characteristics of subjects and clinical outcomes

	**UFT therapy**	**Surgery alone**
*Number of patients*	139	135
Age (median)	59	58
Sex (male)	84	83
		
*Location of the tumour*		
Below the promontrium	43	39
Below the lower edge of the second sacral bone	39	44
Below the rectouterine fossa or rectovesical fossa	58	53
		
*Pathological tumour stage* a		
T1	8	11
T2	21	16
T3	94	91
T4	17	18
		
*Pathological nodal stage* a		
N1	89	90
N2	22	22
N3	29	24
Positive lateral pelvic	11	7
Lymph node		
		
Adverse event more than grade 3	24	5
		
No. of recurrences	32	53
		
	%	%
3-year survival (95% CI)	91 (86–97)	81 (73–88)
3-year relapse-free		
survival (95% CI)	78 (71–86)	60 (51–69)

CI=confidence interval; QALYs=quality-adjusted life-years.

The results are presented according to ITT (intention to treat).

a The 1997 Tumour Node Metastasis (TNM) Classification of Malignant Tumours (International Union Against Cancer).

**Table 2:** Incremental effectiveness and costs of adjuvant UFT therapy (discount rate: 3% for both effectiveness and costs)

**Period**	**UFT therapy**	**Surgery alone**	**Incremental effectiveness and costs (95% CI)**
*(A) Effectiveness and costs*			
	*Life years*		
5.6-year observation	4.89	4.45	0.44 (0.14–0.75)
10-year follow-up	8.06	6.96	1.08 (0.22–1.94)
Over lifetime	16.03	12.93	3.09 (1.29–4.86)
			
	*QALYs*		
5.6-year observation	3.30	2.81	0.50 (0.24–0.76)
10-year follow-up	5.43	4.47	0.96 (0.43–1.47)
Over lifetime	10.68	8.38	2.28 (1.15–3.38)
			
	*Costs ($)*		
5.6-year observation	8742	11 199	−2457 (−4751 to −164)
10-year follow-up	10 037	11 767	−1771 (−4110 to 719)
Over lifetime	10 109	11 921	−1843 (−4208 to 601)
			
	**Incremental cost-effectiveness ratio**		
**Period**	**Cost ($) per LY gained (95% CI)**	**Cost ($) per QALY gained (95% CI)**	
*(B) Cost-effectiveness ratio*			
5.6-year observation	−5559 (−13 254 to −420)	−4969 (−11 057 to −364)	
10-year follow-up	−1573 (−10 188 to 597)	−1815 (−7156 to 634)	
Over lifetime	−594 (−2452 to 169)	−802 (−2899 to 233)	

CI=confidence interval; QALYs=quality-adjusted life-years; UFT=uracil–tegafur.

**Table 3:** Mean costs per patient during observation period (no discounting)

**Item**	**UFT therapy**	**Surgery alone**
Consultation	125 ($)	56 ($)
*Treatment*		
Drugs	3076	NA
Prescription	66	NA
*Tests*		
Diagnostic tests	346	185
Imaging tests	1068	1098
Side effects	2	1
Recurrence	3212	5061
End of life	2131	6226
Total costs	10 026	12 628

NA=not applicable; UFT=uracil–tegafur.

**Table 4:** One-way sensitivity analysis of important factors

**Factor**	**Cost-effectiveness ratio ($ per QALY gained)**
Base case analysis	−4969
	
Recurrence rate (95% CI)	−5304 to −4679
	
Utility (25th, 75th percentile)	
Non-recurrence	−7063 to −4328
Recurrence	−5593 to −4471
	
QALY (95% CI)	−10 434 to −3244
UFT cost (95% CI)	−7506 to −2432
Total cost (95% CI)	−9695 to 334

CI=confidence interval; QALY=quality-adjusted life-year; UFT=uracil–tegafur.

Discount rate: 3% for both cost and effectiveness; period: observation.
